# Association of Parental Height With Offspring Stunting in 14 Low- and Middle-Income Countries

**DOI:** 10.3389/fnut.2021.650976

**Published:** 2021-08-11

**Authors:** Han Wu, Chuanwei Ma, Liu Yang, Bo Xi

**Affiliations:** Department of Epidemiology, School of Public Health, Cheeloo College of Medicine, Shandong University, Jinan, China

**Keywords:** parent, height, stunting, under-five children, low- and middle-income countries

## Abstract

**Background:** Maternal height has been confirmed to be associated with offspring stunting in low- and middle-income countries (LMICs), but only limited studies have examined the paternal-offspring association, and few studies have examined the joint effect of maternal and paternal height on stunting.

**Objective:** To investigate the association between parental height and stunting of children aged under five in LMICs.

**Methods:** We obtained data from the Demographic and Health Surveys (DHS) conducted in 14 LMICs from 2006 to 2016. The association between maternal and paternal height and height-for-age *z* score (HAZ) of children aged under five was analyzed using a linear regression model in consideration of complex survey design, and regression coefficients (β) with 95% confidence intervals (CIs) were reported. Then, the association between maternal and paternal height quintile and child stunting was analyzed using a modified Poisson regression approach with robust error variance in consideration of complex survey design with adjustment for covariates. The effect estimates were expressed as relative risks (RRs) with 95% CIs.

**Results:** A total of 50,372 singleton children were included and the weighted prevalence of stunting was 34.5%. Both maternal height and paternal height were associated with child HAZ (β = 0.047; 95% CI, 0.043, 0.050; and β = 0.022; 95% CI, 0.018, 0.025, respectively). Compared with those born to the tallest mothers and fathers, children from the shortest mothers and the shortest fathers had higher risks of stunting (adjusted RR = 1.89; 95% CI, 1.78, 2.01; adjusted RR = 1.56; 95% CI, 1.47, 1.65, respectively). The mother-offspring associations are substantively larger than the father-offspring associations for each corresponding height quintile. Children from the shortest parents had the highest risk of stunting compared with children from the tallest parents (adjusted RR = 3.23; 95% CI, 2.83, 3.68).

**Conclusions:** Offspring born to short parents are at increased risk of stunting in LMICs, and this intergenerational effect is partly driven by maternal intrauterine influence. This suggests the importance of improving the nutritional status of children and adults in LMICs, especially female caregivers.

## Introduction

The term stunting refers to linear growth retardation and decreased development in children caused by poor nutrition (1). Children are defined as stunted if their height-for-age *z* score (HAZ) is less than two SDs below the WHO child growth standards median (1). Although the global prevalence of stunting among children under 5 years declined from 32.4% in 2000 to 21.3% in 2019, there are still 144 million children who struggle with this disorder (2). Furthermore, stunting disproportionately affects 2.1 million and 147.8 million children in high-income countries (HICs) and low- and middle-income countries (LMICs) in 2018, respectively (3). For stunted children, they may suffer from severe short-term (e.g., delayed motor and cognitive development), medium-term (e.g., poorer school performances), and long-term consequences (e.g., reduced stature and earnings, and increased risk of non-communicable chronic diseases in adulthood) (4).

Existing studies have revealed a variety of influencing factors such as, genetic disposition, household wealth, parental education, smoking, and sanitation, that can predispose individuals to the development of stunting (5, 6). Several recent studies further compared the magnitude between maternal and paternal influences, which generally underscored the relative importance of maternal roles for child development and health. For instance, a study of children aged 36–59 months from multiple LMICs suggested that the associations between maternal education and child HAZ (primary: β = 0.17, 95% confidence interval (CI): 0.14, 0.20; secondary or higher: β = 0.37, 95% CI: 0.33, 0.41, respectively) were much larger than those of paternal education and HAZ (primary: β = 0.11, 95% CI: 0.08, 0.15; secondary or higher: β = 0.20, 95% CI: 0.16, 0.24) (7). The UN Children's Fund (UNICEF) maternal and child health survey data of China revealed that, compared with children who were cared for primarily by their mothers, children cared for by the fathers had an increased risk of being stunted and were less likely to receive age-appropriate breastfeeding and minimum acceptable diet (8). Results from the Avon Longitudinal Study of Parents and Children showed that maternal smoking had a much larger effect on child height (β = −0.68, 95% CI: −1.02, −0.33) than paternal smoking (β = 0.04, 95% CI: −0.26, 0.33) (9).

Some research focused on the intergenerational effect by parental height, which captures information on the genetic potential of the offspring, the shared environment of the parents, and the health stock accumulated through their life course, especially the environmental condition in their early childhood (10). In addition, the association between maternal height and children stunting could additionally reflect the intrauterine effects as fathers do not experience pregnancy (11). Stronger maternal-offspring than paternal-offspring associations would imply the existence of intrauterine effects, whereas similar maternal and paternal associations with offspring stunting would suggest predominantly genetic and environmental effects (12). However, maternal height has been repeatedly reported to associate with offspring stunting in LMICs (13–16), but only limited studies have examined the paternal-offspring association (17), which impedes the assessment of intrauterine influence. Furthermore, few studies have examined the joint effect of maternal and paternal height on offspring stunting.

We hypothesized that there might be an intrauterine effect of maternal height on child stunting, and a child born to the shortest mother and father might have the highest risk of being stunted. In addition, some existing studies found that there was a sex-specific or age-specific parental-offspring association (18–20), whereas some others did not (21). Therefore, we also attempted to explore such associations based on much larger sample size. In this study, we used data covering a large nationally representative sample of children who were aged under five from 14 LMICs to examine the associations between maternal and paternal height and offspring HAZ and stunting.

## Methods

### Study Design and Population

Data were obtained from the Demographic and Health Surveys (DHS) conducted in 14 LMICs from 2006 to 2016 (for a full list of countries, as shown in Supplementary Table 1). The DHS are nationally representative household sample surveys and they collect health and nutrition data for women of reproductive age (15–49 years), their children (0–59 months), and their households. DHS are cross-sectional surveys and detailed information on the DHS has been described elsewhere (22). Anthropometry data were available for women and their children across most standard DHS surveys. In some of these surveys, households that were selected to contribute information to the woman and child health survey were also eligible for random selection into a subsample for collecting anthropometry data for men. Until January 2021, only a total of 14 out of 100 s DHS surveys collected anthropometry data not only for children and women but also for men. In the present study, we included data from these surveys to generate a pooled dataset containing data of singleton children aged under five and their parents. In these surveys, height for women, men, and live offspring were measured at the same visit (during the survey) by trained examiners according to a standardized protocol by using a calibrated adjustable board (accurate to 0.1 cm).

The DHS data-collection procedures were approved by ICF institutional review board and by the relevant human subject committees in each country. Oral informed consent was obtained from each of the survey participants. The present study is exempt from a full review of the institution of the authors because of the use of an anonymized public-use dataset.

### Outcomes, Exposures, and Covariates

The stunting of children aged under five was the outcome of interest. HAZ was first calculated by subtracting the height of a child from the median height for a child of that age and sex and dividing by the SD of the height for a child of that age and sex in the WHO reference population (23). Then, stunting was defined as a HAZ less than two SD of the median (23).

Maternal and paternal height were the exposure of interest. Adult height <120 cm or larger than 220 cm was considered to be biological implausible (24). To avoid the heterogeneity of adult height across countries, maternal and paternal heights were classified according to their height quintile, respectively, which were calculated based on the samples from the original population within each country. Mothers and fathers assigned to the fifth quintile were considered to be of high stature, and they had the smallest probability of having a stunted offspring. They served as a reference for comparison.

Covariates included in the main analyses were maternal age, parental age, the maternal highest level of education, and the paternal highest level of education; sex and age of the child, birth interval, and birth order; household wealth quintile (first quintile: poorest to the fifth quintile: richest, within each country), residence (urban or rural), country; and survey year. Parental smoking status, parental occupations, breastfeeding initiation time and duration time, child anemia, child acute respiratory infection (ARI), and diarrhea within 2 weeks preceding the survey, the household drinking water source was also important covariates, but they were not consistently collected or have substantial missingness in several countries, and thus, they were further adjusted in the sensitivity analyses. The DHS provides a wealth quintile for each household by evaluating standard household assets and durables, which is a relative index and a reliable proxy for household economic status (6). Parental smoking status was dichotomized (yes or no) to define whether the mother or father was a current smoker (use of smoked tobacco or smokeless tobacco) (25). Parental occupation was categorized as not working, non-manual, manual, and agricultural. Breastfeeding initiation time was categorized as the initiation of breastfeeding within 1 h of birth and >1 h of birth (5). Breastfeeding duration time was categorized as breastfeeding duration <1, 1, 2 years or more, and never breastfed. Anemia was defined as the concentration of blood hemoglobin (detailed information about hemoglobin testing is on https://dhsprogram.com/topics/Anemia.cfm) <110 g/L, and the severity of anemia for children aged 6–59 months was classified as mild (100–109 g/L), moderate (70–99 g/L), and severe (< 70 g/L) according to recommendations from the WHO (26). ARI was defined as coughing accompanied by short, rapid breathing within 2 weeks preceding the survey, and these self-reported symptoms have been used in some previous studies to indicate the onset of ARI (27). Diarrhea occurrence of the child within 2 weeks preceding the survey was reported by the mother and this variable was dichotomized (yes or no). The drinking water source was dichotomized into “improved” (such as water from a borehole, water from a protected spring or well, rainwater, and bottled water) and “unimproved” (such as water from unprotected dug wells or springs, water from a vendor or tanker-truck, and surface water) according to the WHO/UNICEF JMP for water supply and sanitation definition (28).

### Statistical Analysis

The DHS uses a multistage stratified sampling design to collect data, and each survey was stratified by residence (urban and rural) and then further by country-specific geographic or administrative areas. To adjust for the complex survey design of the DHS, new indicators for cluster and strata were generated in consideration of “country” variables (to make them unique in the pooled dataset) based on the original cluster and strata. In addition, sampling weights were rescaled by equal proportional weighting, which could account for arbitrary differences in sample sizes across countries and reduce the influence of larger survey samples (such as those from India). Detailed information about this process is presented in the Supplementary Material (page 2).

We undertook a complete case analysis. Multiple imputation method was not performed because of the heterogeneous study settings across countries. Although the subsample of men and women selected for anthropometry were randomly selected (29), a large proportion (84%) of the participants were not included in the analysis mainly because of data missingness. To compare the characteristics between the included and excluded participants, we examined the difference of child sex, child age, maternal age, and paternal age between the two groups for each country, respectively.

Linear regression models in consideration of complex survey design were used to estimate the association between parental height and child HAZ. The association was expressed as regression coefficients (β) with a 95% CI. As the prevalence of stunting was more than 10%, using the odds ratio estimated from a conventional logistic regression model to represent relative risk (RR) directly was problematic (30). Thus, we used a modified Poisson regression approach with robust error variance in consideration of complex survey design (using new indicators for the cluster, strata, and sampling weights) to estimate the association between parental height quintile and stunting with adjustment for other covariates (30). In this method, the association was expressed as RR with 95% CI. A detailed introduction about modified Poisson regression is presented in the Supplementary Material (page 3).

We conducted subgroup analyses by stratifying children by sex and age (1–2 and 3–4 years). Infants aged <1 year were not included for subgroup analysis because stunting may not fully be a reliable indicator for them when infants are still developing. We also conducted several sensitivity analyses to test the robustness of the findings. As mentioned above, covariates including maternal age, the maternal highest level of education, paternal age, and the paternal highest level of education; sex and age of the child, birth interval, and birth order; household wealth status, residence, country, and survey year have already been adjusted in the main model. For each sensitivity analysis, except for all the covariates from the main model, we then additionally adjusted for parental smoking status and occupations, breastfeeding status, child anemia, child ARI, child diarrhea, and household drinking water source separately. We further adjusted for these variables and all the covariates from the main model simultaneously. We also reported results for two groups of the country, one group including samples only from India and the other including samples from non-Indian countries, as samples from India accounted for 58% of the total sample size. In addition, we also restricted the samples to only the first birth offspring. To test the influence of non-random missingness on the results, we included the samples only from countries where the characteristics between the included and excluded participants were not significantly different.

All statistical analyses were performed using SAS version 9.4. A two-sided *p* < 0.05 was considered statistically significant.

## Results

### Study Population and Characteristics

The participant flowchart is presented in Supplementary Figure 1. In this study, we analyzed data from a total of 50,372 singleton children under five from 14 LMICs, among whom there were 17,432 (34.5%) stunted children. Table 1 presents the percentage of children and weighted prevalence of stunting according to sociodemographic characteristics. A total of 25,810 (51.2%) were boys and 14,649 (29.1%) were first-birth children. The mean of child HAZ, maternal height, and paternal height were −1.37 (SD: 1.72), 154.3 (SD:6.8) cm, and 165.7 (SD:7.4) cm, respectively (Supplementary Table 1). Children born to the tallest mothers and fathers had the lowest occurrence of offspring stunting, and the prevalence decreased as maternal and paternal height increased. The prevalence of stunting varied among countries from 16.4% in the Maldives to 37.4% in Ethiopia (Supplementary Table 1). The characteristics (child sex, child age, maternal age, and paternal age) between the included and excluded participants were not significantly different in 10 countries (Supplementary Table 2).

**Table 1:** Percentage of children and weighted prevalence of stunting according to sociodemographic characteristics.

	**Children observed**		**Stunting**	
	***n***	**Percentage, %**	***n***	**Weighted prevalence, %**
Total	50,372	100.0	17,432	34.5
**Maternal**				
**Age, years**				
<20	1,509	3.0	481	32.2
20–29	30,454	60.5	10,808	35.0
30–39	16,029	31.8	5,325	33.7
≥40	2,380	4.7	818	34.9
**Highest level of education**				
None	16,603	33.0	7,134	43.8
Primary	11,054	21.9	3,896	35.3
Secondary or higher	22,715	45.1	6,402	27.4
**Height quintile (within country)**				
First, short	10,362	20.6	5,057	48.4
Second	10,309	20.5	3,958	38.2
Third	10,165	20.2	3,457	34.2
Fourth	10,020	19.9	2,844	28.1
Fifth, tall	9,516	18.9	2,116	21.4
**Paternal**				
**Age, years**				
<20	137	0.3	41	27.5
20–29	16,252	32.3	5,916	36.0
30–39	24,236	48.1	8,197	33.7
≥40	9,747	19.4	3,278	34.1
**Highest level of education**				
None	10,856	21.6	4,674	43.7
Primary	11,493	22.8	4,187	36.0
Secondary or higher	28,023	55.6	8,571	30.1
**Height quintile (within country)**				
First, short	9,910	19.7	4,478	45.3
Second	10,256	20.4	4,008	39.2
Third	10,209	20.3	3,504	34.0
Fourth	10,390	20.6	3,145	30.6
Fifth, tall	9,607	19.1	2,297	23.2
**Child**				
**Sex**				
Male	25,810	51.2	9,209	35.7
Female	24,562	48.8	8,223	33.3
**Birth interval**				
First child	14,649	29.1	4,636	30.9
≤ 23 months	7,937	15.8	3,373	42.1
24–47 months	18,316	36.4	6,738	37.0
≥48 months	9,470	18.8	2,685	29.0
**Birth order**				
First	14,649	29.1	4,636	30.9
Second	13,601	27.0	4,547	33.8
Third	8,513	16.9	3,124	36.8
Fourth	5,213	10.3	1,971	37.6
≥Fifth	8,396	16.7	3,154	37.8
**Household**				
**Wealth quintile, within country**				
First, poorest	13,052	25.9	5,684	44.9
Second	11,437	22.7	4,468	39.6
Third	10,044	19.9	3,326	33.5
Fourth	8,517	16.9	2,441	29.1
Fifth, richest	7,322	14.5	1,513	19.6
**Location**				
Urban	12,920	25.6	3,663	27.0
Rural	37,452	74.4	13,769	37.4

### Association Between Parental Height and Stunting

Table 2 presents the unadjusted and adjusted β and RRs with 95% CIs between parental height and HAZ, and parental height quintile and stunting, respectively. Both maternal height and paternal height were associated with child HAZ and the mother-offspring association is substantively larger than the father-offspring association (β = 0.047; 95% CI, 0.043, 0.050; and β = 0.022; 95% CI, 0.018, 0.025, respectively). Children from the shortest mothers had an 89% higher risk of stunting compared with those from the tallest women (adjusted RR = 1.89; 95% CI, 1.78, 2.01). Similarly, children from the shortest fathers had a 56% higher risk of stunting compared with those from the tallest men (adjusted RR = 1.56; 95% CI, 1.47, 1.65). Moreover, the mother-offspring associations are also larger than the father-offspring associations for each corresponding height quintile.

**Table 2:** Unadjusted and adjusted β between maternal and paternal height and child height-for-age z score (HAZ) and relative risk (RR) between maternal and paternal height quintile and child stunting with their 95% confidence intervals (CIs).

**Exposure**	**Unadjusted**					Adjusted^*^			
	**Maternal**		**Paternal**		**Maternal**			**Paternal**	
	**β/RR (95% CI)**	***P* Value**	**β/RR (95% CI)**	***P* Value**	**β/RR (95% CI)**	***P* Value**		**β/RR (95% CI)**	***P* Value**
Height	0.043 (0.040–0.047)	<0.001	0.022 (0.019–0.025)	<0.001	0.047 (0.043–0.051)	<0.001		0.022 (0.018–0.025)	<0.001
**Height quintile (within country)**									
First, short	2.06 (1.94–2.19)	<0.001	1.89 (1.78–2.01)	<0.001	1.88 (1.77–1.99)	<0.001		1.56 (1.47–1.65)	<0.001
Second	1.66 (1.56–1.77)	<0.001	1.58 (1.48–1.68)	<0.001	1.57 (1.47–1.67)	<0.001		1.44 (1.35–1.53)	<0.001
Third	1.52 (1.43–1.62)	<0.001	1.46 (1.37–1.56)	<0.001	1.46 (1.37–1.56)	<0.001		1.30 (1.22–1.39)	<0.001
Fourth	1.27 (1.18–1.36)	<0.001	1.25 (1.17–1.34)	<0.001	1.24 (1.16–1.33)	<0.001		1.21 (1.13–1.29)	<0.001
Fifth, tall	1.00 (Reference)		1.00 (Reference)		1.00 (Reference)			1.00 (Reference)	
P for trend		<0.001		<0.001		<0.001			<0.001

* *Models were adjusted for maternal age, the maternal highest level of education, paternal age, and paternal highest level of education; sex and age of the child, birth interval, and birth order; household wealth status, residence, country, and survey year*.

**Figure 1:**
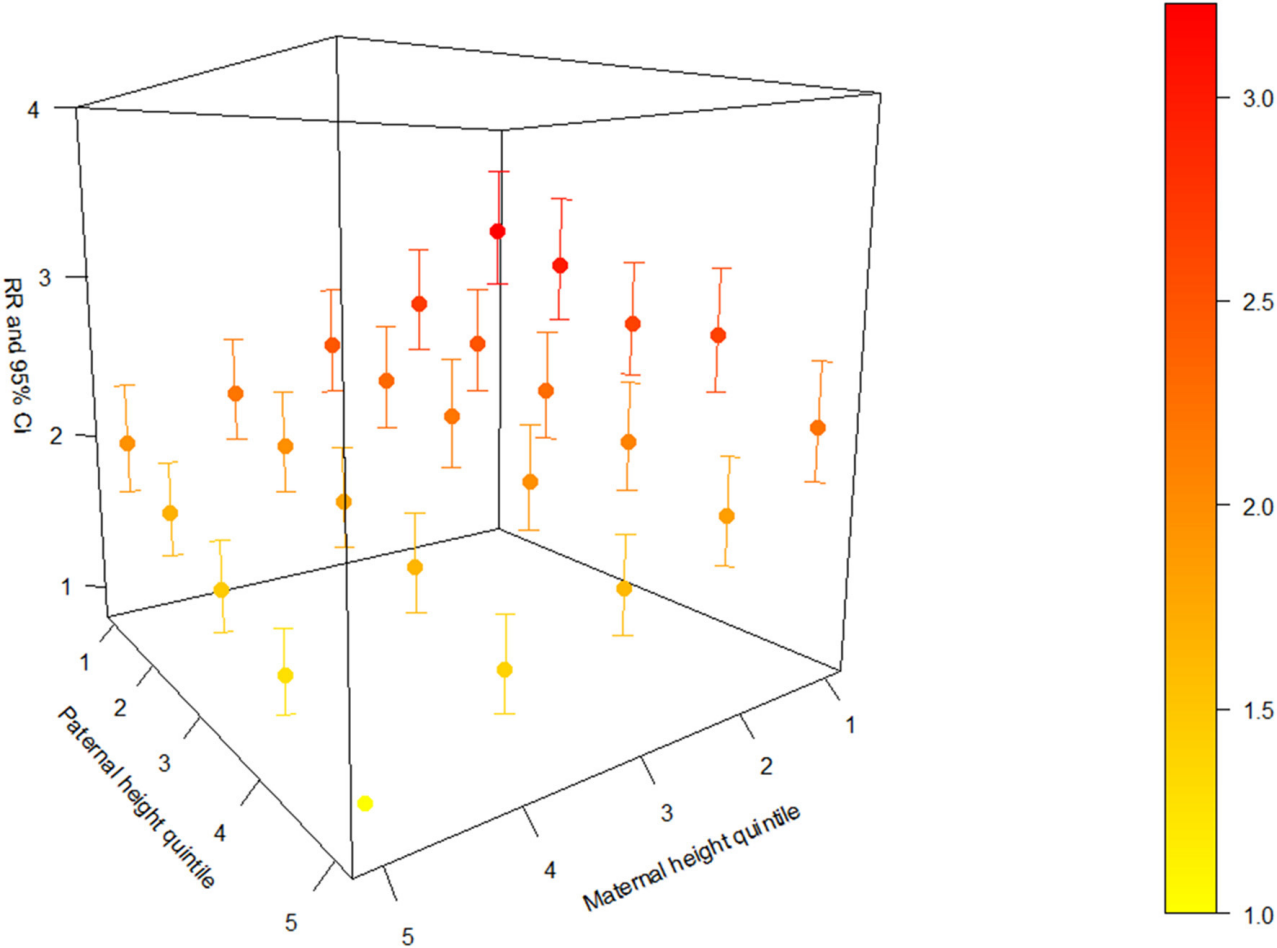
Adjusted relative risk (RR) with a 95% confidence interval (CI) based on each combination of maternal and paternal height quintile.

To present the joint effect between maternal and paternal height for offspring stunting, we used a 3-D plot to show the adjusted RRs with 95% CIs based on each combination of maternal and paternal height quintile (Figure 1). There was an increasing trend of the adjusted effect estimates as maternal and paternal height quintiles decrease, and children from the shortest parents had about three times the risk of stunting compared with children from the tallest parents (adjusted RR = 3.23; 95% CI, 2.83, 3.68). The values of the adjusted RRs with 95% CIs are presented in Supplementary Table 3.

### Subgroup and Sensitivity Analyses

In subgroup analyses (Table 3), the adjusted associations by offspring sex and age showed similar patterns. However, the association between maternal height and offspring HAZ and maternal height quintile and stunting were larger for girls than boys, whereas the father-offspring associations were slightly larger for boys than girls. In addition, the associations between both maternal and paternal height and offspring stunting were smaller for children aged 1–2 years than children aged 3–4 years. In sensitivity analyses (Supplementary Table 4), the adjusted associations also showed similar patterns compared with the main results after adjustment for a variety of additional covariates, after including samples from India, samples from non-Indian countries, samples of the first birth offspring, and samples from countries where the characteristics between the included and excluded participants were not significantly different, respectively.

**Table 3:** Unadjusted and adjusted β between maternal and paternal height and child HAZ and RR between maternal and paternal height quintile and children stunting with their 95% CIs by child sex and age.

**Group**	**Unadjusted**					Adjusted^*^					
	**Maternal**			**Paternal**			**Maternal**			**Paternal**	
	**β/RR (95% CI)**	***P* Value**		**β/RR (95% CI)**	***P* Value**		**β/RR (95% CI)**	***P* Value**		**β/RR (95% CI)**	***P* Value**
**Offspring sex**											
**Boys (** ***n*** **=** **25,810)**
Height	0.042 (0.038–0.047)	<0.001		0.025 (0.021–0.029)	<0.001		0.044 (0.040–0.049)	<0.001		0.024 (0.020–0.028)	<0.001
**Height quintile (within country)**											
First, short	1.95 (1.80–2.11)	<0.001		1.78 (1.64–1.92)	<0.001		1.77 (1.64–1.92)	<0.001		1.60 (1.49–1.73)	<0.001
Second	1.60 (1.47–1.74)	<0.001		1.59 (1.46–1.72)	<0.001		1.51 (1.39–1.64)	<0.001		1.46 (1.35–1.58)	<0.001
Third	1.46 (1.35–1.59)	<0.001		1.39 (1.28–1.51)	<0.001		1.41 (1.30–1.53)	<0.001		1.32 (1.22–1.43)	<0.001
Fourth	1.22 (1.11–1.34)	<0.001		1.30 (1.20–1.42)	<0.001		1.19 (1.09–1.31)	<0.001		1.25 (1.15–1.36)	<0.001
Fifth, tall	1.00 (Reference)			1.00 (Reference)			1.00 (Reference)			1.00 (Reference)	
P for trend		<0.001			<0.001			<0.001			<0.001
**Girls (** ***n*** **=** **24,562)**											
Height	0.044 (0.039–0.050)	<0.001		0.019 (0.014–0.024)	<0.001		0.050 (0.044–0.056)	<0.001		0.019 (0.014–0.024)	<0.001
**Height quintile (within country)**											
First, short	2.21 (2.02–2.42)	<0.001		1.67 (1.53–1.83)	<0.001		2.02 (1.84–2.21)	<0.001		1.50 (1.38–1.64)	<0.001
Second	1.75 (1.60–1.93)	<0.001		1.54 (1.41–1.68)	<0.001		1.65 (1.50–1.81)	<0.001		1.42 (1.30–1.56)	<0.001
Third	1.59 (1.45–1.76)	<0.001		1.37 (1.25–1.51)	<0.001		1.53 (1.39–1.68)	<0.001		1.30 (1.18–1.42)	<0.001
Fourth	1.33 (1.19–1.47)	<0.001		1.23 (1.12–1.35)	<0.001		1.31 (1.19–1.45)	<0.001		1.17 (1.06–1.28)	0.001
Fifth, tall	1.00 (Reference)			1.00 (Reference)			1.00 (Reference)			1.00 (Reference)	
P for trend		<0.001			<0.001			<0.001			<0.001
**Offspring age**											
**1 to 2 years (** ***n*** **=** **20,370)**
Height	0.044 (0.038–0.049)	<0.001		0.018 (0.012–0.023)	<0.001		0.042 (0.037–0.047)	<0.001		0.021 (0.017–0.026)	<0.001
**Height quintile (within country)**											
First, short	2.02 (1.86–2.19)	<0.001		1.69 (1.56–1.83)	<0.001		1.88 (1.73–2.04)	<0.001		1.52 (1.40–1.65)	<0.001
Second	1.58 (1.45–1.73)	<0.001		1.51 (1.39–1.65)	<0.001		1.51 (1.39–1.65)	<0.001		1.40 (1.29–1.53)	<0.001
Third	1.49 (1.36–1.62)	<0.001		1.37 (1.25–1.49)	<0.001		1.44 (1.32–1.57)	<0.001		1.29 (1.18–1.40)	<0.001
Fourth	1.25 (1.14–1.37)	<0.001		1.25 (1.14–1.36)	<0.001		1.24 (1.13–1.36)	<0.001		1.18 (1.09–1.29)	<0.001
Fifth, tall	1.00 (Reference)			1.00 (Reference)			1.00 (Reference)			1.00 (Reference)	
P for trend		<0.001			<0.001			<0.001			<0.001
**3 to 4 years (** ***n*** **=** **20,424)**											
Height	0.051 (0.045–0.057)	<0.001		0.019 (0.014–0.024)	<0.001		0.048 (0.043–0.053)	<0.001		0.025 (0.020–0.030)	<0.001
**Height quintile (within country)**											
First, short	2.16 (1.97–2.38)	<0.001		1.77 (1.61–1.93)	<0.001		1.93 (1.76–2.11)	<0.001		1.58 (1.44–1.72)	<0.001
Second	1.83 (1.66–2.02)	<0.001		1.64 (1.50–1.80)	<0.001		1.70 (1.54–1.87)	<0.001		1.50 (1.37–1.64)	<0.001
Third	1.58 (1.43–1.74)	<0.001		1.43 (1.30–1.58)	<0.001		1.50 (1.36–1.65)	<0.001		1.35 (1.23–1.48)	<0.001
Fourth	1.35 (1.22–1.49)	<0.001		1.34 (1.21–1.47)	<0.001		1.31 (1.19–1.44)	<0.001		1.26 (1.15–1.39)	<0.001
Fifth, tall	1.00 (Reference)			1.00 (Reference)			1.00 (Reference)			1.00 (Reference)	
P for trend		<0.001			<0.001			<0.001			<0.001

* *Models were adjusted for maternal age, the maternal highest level of education, paternal age, and paternal highest level of education; sex of the child (only in subgroups by children age), child age, birth interval, and birth order; household wealth status, residence, country, and survey year*.

## Discussion

Using data of more than 50,000 samples from 14 LMICs, we found a clear relationship between parental height and risk of offspring stunting. The risk of stunting increases as maternal and paternal height decreases, and children from the shortest parents had the highest risk of stunting. The larger association between maternal height and child HAZ and stunting than paternal-offspring association in magnitude suggests that the intergenerational effect is partly driven by maternal intrauterine influences.

The findings, especially the association between maternal height and child HAZ and the association between maternal height and stunting, were consistent with most previous studies (13–16). Other studies [from the Czech Republic (31), Brazil (32), and Vietnam (33)] using HAZ as an outcome of interest also found that higher parental height was associated with higher HAZ among young children, which was similar to the findings. The difference between our study and these previous studies lies in the inclusion of paternal height. Only one study from India reported that both higher maternal and paternal height were associated with reduced risk of stunting, and the maternal-offspring association was also larger than the paternal-offspring association (17).

There might be several mechanisms explaining the association between parental height and offspring stunting. First, the intergenerational associations may reflect genetic mechanisms transferring endowments from parents to their children (13). Several chromosomes (such as the 7th, 8th, 20th, and sex chromosomes) have been implicated in the development of human height; thus, both mothers and fathers may pass on height-determining genes to their offspring (18, 34, 35). The subgroup results by offspring sex showed that the mother-daughter association was larger than the mother-son association in magnitude. Kelly et al. (18) reported similar findings as they found that the correlation between maternal height and height of the girl was stronger than the correlation between maternal height and height of the boy at the age of five. Therefore, the growth control gene located on the sex chromosomes may be linked to the above sex-specific association (18).

Second, the intergenerational associations may also be driven by parental health and the ability of investments made in child health during the prenatal and childhood periods (19). For example, shorter parents are more likely to be unhealthy and are too sick to spend adequate time and effort on health-promoting activities for their children (13). In addition, they may employ themselves in relatively low reward occupations, and this may subsequently lead to under-investment in the human capital of their offspring (19). Therefore, their children will receive a poor-quality and low-diversity diet. The subgroup results by children age reflect this point: both of the maternal-offspring and paternal-offspring associations in older children are larger than those in younger children in magnitude, presumably because of shared dietary environments.

It is of note that the results of the authors indicated the existence of the maternal intrauterine effect. Several studies focusing on the parental-offspring weight status relationship also reported that maternal-offspring association was larger than paternal-offspring association (18, 36). In children born following egg donation, birth weight was more closely associated with the height of the recipient than the height of the donor, also implying a maternal intrauterine effect (37). This effect can be interpreted by biomechanical and biological mechanisms. Shorter mothers generally have narrower pelves which leads to a suboptimum uterine environment for fetal growth (38). Maternal short height is also an indicator of her lower health stock and inadequate net nutrition accumulation (10). Lack of nutrition of pregnant women adversely affects placental growth that causes inadequate nutrient transmission and oxidative stress to the fetus (39, 40), which may subsequently trigger epigenetic modification such as DNA methylation to affect fetal programming that leads to fetal growth faltering (41). All these above conditions can increase the risk of delivering infants with low birth weight and immature immune systems, who are susceptible to infection (42). Then, the infection may predispose children to mucosal damage and inadequate absorption of essential nutrients (43). However, the observed larger association between maternal height and child HAZ and stunting than paternal-offspring associations in magnitude may also indicate behavioral and social paths related to gender norms and roles. This is because mothers generally have greater caregiving roles than fathers, and taller mothers may have great aspirations for their children to be healthier and then provide more nutritious foods and optimal feeding practices (8). Conversely, fathers may have less direct roles in their lives, and they would exert less influence on parenting children (44).

The paternal height still has its independent effect on child stunting, although the association in magnitude is weaker than the maternal-offspring association. In addition to the mechanisms of paternal effect introduced above, there is growing evidence that paternal nutrition is associated with offspring health. Numerous animal models have confirmed that poorer paternal diet, such as low-protein and life-long folate deficiency, can lead to worse sperm quality (45). Some human epidemiological data revealed that male low sperm quality is associated with increased risk of offspring low birth weight (46), which is a known risk factor for child stunting (47). Therefore, improving the nutrition of male adults may also generate a positive influence on reducing child stunting in LMICs.

In the past few years, there has been virtually no change in mean population height in some LMICs (especially in some African and south and southeast Asian countries), and there was not an evident reduction trend of short stature prevalence in LMICs (48). Previous studies have shown that nutritional supplementation could improve birth weight in malnourished women (49). By contrast, there is less effect on birth weight for marginally malnourished women, and there is no effect on birth weight in well-nourished women (49). Thus, we presume that nutrition intervention programs targeting high burden regions where the short stature of the population are more prevalent would result in a greater effect on reducing child stunting and probably an increased cost-effectiveness than untargeted intervention coverage. Therefore, policymakers should consider a precision public health approach instead of the existing global “one-size-fits all” guideline (50). We argue that a segmented, targeted, and equity-focused approach may be more impactful and would potentially be more cost-effective than a population approach (51). For example, countries with a relatively high prevalence of adult short stature (generally defined with the height of an individual between 145 and 155 cm) or child stunting should consider a subnational geographic nutrition intervention program targeting adults with short stature or stunted children and provide them with sufficient energy and protein supplements (51). Future research should identify appropriate cutoffs for the prevalence of adult short stature and child stunting to design this kind of geographic-specific intervention program, as guidance on this is limited.

The study has several strengths. First, the study was based on a large and nationally representative sample in LMICs, and the anthropometry data are reliable because of the standardized protocols and accurate measurements. Second, we examined the intergenerational effect of parental height after adjusting for well-established determinants of children stunting, such as parental age and birth history, and social factors such as parental education, household wealth, and residence (5). In addition, the relationship was robust across sensitivity analyses after additional adjustment for parental smoking, parental occupation, and breastfeeding initiation time. However, several limitations should be considered. First, we did not control previously established immediate causes of children stunting, such as poor dietary diversity and parasital infection in the study (5). Second, only 14 out of more than 90 LMICs were included in the analysis of the authors because there was no male anthropometry data in most countries in current DHS datasets. Third, access to quality health care could also affect the health of both parents and the offspring. Thus, the results may be biased toward more underprivileged populations in LMICs where people have less access to quality healthcare services compared with their counterparts in HICs. Therefore, the significant results of the study should be interpreted with caution. Fourth, a large proportion of data of the participants were missing and, thus, excluded from the analysis, which may cause potential selection bias. However, the results of sensitivity analysis, including the samples only from countries where the characteristics between the included and excluded participants were not significantly different, showed similar patterns with the main results. Therefore, this may have a negligible influence on the results.

## Conclusions

In conclusion, the study demonstrates that offspring born to short parents are at an increased risk of stunting in LMICs, and this intergenerational effect is partly driven by the maternal intrauterine effect. Findings from this study suggest the importance of improving the nutritional status of children and adults in LMICs, especially women caregivers. Nutrition intervention programs are warranted for both adults and children, as adequate nutrition can help achieve optimum height in adulthood and also have long-term consequences on offspring growth.

## Data Availability Statement

Publicly available datasets were analyzed in this study. This data can be found at: https://dhsprogram.com/data/available-datasets.cfm.

## Ethics Statement

The studies involving human participants were reviewed and approved by Inner City Fund (ICF) institutional review board. Written informed consent for participation was not provided by the participants' legal guardians/next of kin because: We used a anonymised public-use dataset from Demographic and Health Surveys (DHS), and oral informed consent was obtained from each of the survey participants or their parents according to DHS publisher.

## Author Contributions

HW and BX: conceptualization. CM: methodology and software. LY: validation and visualization. HW and CM: formal analysis and writing the original draft preparation. BX: data curation and supervision. LY and BX: writing the review and editing. All authors contributed to the article and approved the submitted version.

## Conflict of Interest

The authors declare that the research was conducted in the absence of any commercial or financial relationships that could be construed as a potential conflict of interest.

## Publisher's Note

All claims expressed in this article are solely those of the authors and do not necessarily represent those of their affiliated organizations, or those of the publisher, the editors and the reviewers. Any product that may be evaluated in this article, or claim that may be made by its manufacturer, is not guaranteed or endorsed by the publisher.

## Supplementary Material

Supplementary Figure 1 The participant flowchart.

Supplementary Table 1 Sample size, child height-for-age z score (HAZ), maternal height, paternal height, and weighted prevalence of children stunting included for analysis by country.

Supplementary Table 2 Characteristics between the included and excluded participants by country.

Supplementary Table 3 The adjusted relative risk (RR) with 95% confidence intervals (CIs) is based on each combination of maternal and paternal height quintile.

Supplementary Table 4 Adjusted β between maternal and paternal height and child HAZ and RR between maternal and paternal height quintile and children stunting with their 95% CIs for sensitivity analysis.
