# Supplementary figures and images for: Association of Parental Height With Offspring Stunting in 14 Low- and Middle-Income Countries
Source: Front Nutr. 2021 Aug 11;8:650976. doi: 10.3389/fnut.2021.650976 (PMC8384954; doi:10.3389/fnut.2021.650976)

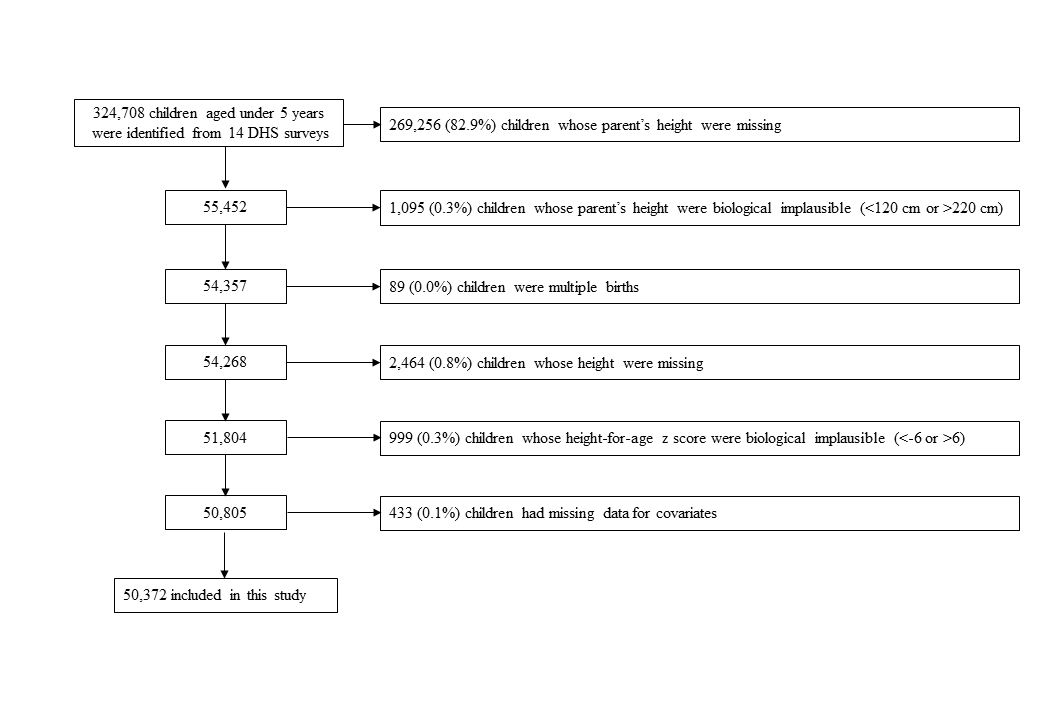

Supplement: Supplementary Figure 1 — The participant flowchart. [file Image_1.TIF]
