# Supplementary material for: Association of Parental Height With Offspring Stunting in 14 Low- and Middle-Income Countries
Source: Front Nutr. 2021 Aug 11;8:650976. doi: 10.3389/fnut.2021.650976 (PMC8384954; doi:10.3389/fnut.2021.650976)
Supplement: Supplementary Table 1 — Sample size, child height-for-age z score (HAZ), maternal height, paternal height, and weighted prevalence of children stunting included for analysis by country. [file Data_Sheet_1.doc]

**Supplementary Materials for:**

**Association of parental height with offspring stunting in 14 low- and middle-income countries**

**Additional information about re-generating PSUs, strata, and sampling weights …… ……p2**

Additional information about the modified Poisson regression **…… …… …… …… …… p3**

Table S1 **…… …… …… …… …… …… …… …… …… …… …… …… …… …… …p4**

Table S2 **…… …… …… …… …… …… …… …… …… …… …… …… …… …… …p5**

Table S3 **…… …… …… …… …… …… …… …… …… …… …… …… …… …… …p7**

Table S4 **…… …… …… …… …… …… …… …… …… …… …… …… …… …… …p8**

**Additional information about re-generating PSUs, strata, and sampling weights**

We pooled the data across all surveys by creating unique identifiers for primary sampling units (PSUs) and strata across all surveys and re-normalized the weights for each survey. First, we generated a new numeric variable for each survey, which varied from 101 to 114 (representing the included 14 surveys respectively) to keep the variable length equal. Second, we combined survey values with the existing PSU and strata values, in order to renumbering them and to make all the PSUs and strata unique in the pooled dataset. At last, sampling weights were rescaled by equal proportional weighting which could account for arbitrary differences in sample sizes across countries and reduce the influence of larger survey samples (such as those from India).

Reference

Bhatta DN, Glantz S. Parental tobacco use and child death: analysis of data from demographic and health surveys from South and South East Asian countries. Int J Epidemiol. 2018;48(1):199-206.

Fahad R, et al. Prevalence of Body Mass Index Lower Than 16 Among Women in Low- and Middle-Income Countries. JAMA. 2015; 314(20):2164-2171.

**Additional information about the modified Poisson regression**

Logistic regression is the most widely-used modeling approach for studying associations between exposures and binary outcomes with adjustment for covariates. For rare events, the odds ratio (OR) estimated from a logistic regression model provides a reasonable approximation of the risk ratio (RR). However, when the outcome is common (the prevalence >10%), the OR will exaggerate the RR. Many literatures have discussed this issue (Viera AJ 2008, McNutt LA, et al 2003).

Conventional Poisson regression has been proposed for analyzing data with rare events when subjects are followed for a variable length of time. But the error for the estimated RR will be overestimated when Poisson regression is applied to binomial data. However, this problem can be rectified by using a robust error variance procedure known as sandwich estimation, thus leading to a technique that referred as a modified Poisson regression with robust error variance. The main advantage of the modified Poisson regression with robust error variance is that it directly estimates the RR rather than the OR. This can be implemented in SAS using SAS PROC GENMOD procedure with the REPEATED statement. An example code is listed in the following:

**proc** **genmod** data = mydata;

class strata psu;

model outcome = exposure covariate1 covariate2 … / dist = poisson link = log;

repeated subject = strata(psu);

weight weight;

**run**;

Reference

McNutt LA, et al. Estimating the relative risk in cohort studies and clinical trials of common outcomes. Am J Epidemiol. 2003;157(10):940-943.

Viera AJ. Odds ratios and risk ratios: what's the difference and why does it matter? South Med J. 2008;101(7):730-734.

Zou G. A modified poisson regression approach to prospective studies with binary data. Am J Epidemiol. 2004;159(7):702-706.

**Table S1**. Sample size, child HAZ, maternal height, paternal height, and weighted prevalence of children stunting included for analysis by country.

| Country | Year | Sample size | Child HAZ | Maternal height | Paternal height | Stunting | |
| --- | --- | --- | --- | --- | --- | --- | --- |
|  |  |  |  |  |  | n | Weighted % |
| Ethiopia | 2016 | 5,457 | -1.35±1.74 | 157.6±6.3 | 169.2±6.8 | 1,970 | 37.4 |
| Ghana | 2014 | 1,560 | -0.94±1.30 | 159.1±5.7 | 170.4±6.5 | 285 | 17.2 |
| India | 2015 | 29,478 | -1.40±1.77 | 151.8±5.9 | 163.5±6.8 | 11,087 | 37.3 |
| Liberia | 2013 | 1,683 | -1.27±1.61 | 156.7±6.0 | 166.7±6.6 | 525 | 28.7 |
| Lesotho | 2014 | 396 | -1.64±1.27 | 156.9±5.7 | 168.1±6.6 | 153 | 34.9 |
| Maldives | 2016 | 861 | -0.87±1.22 | 152.6±5.3 | 164.7±6.2 | 128 | 16.4 |
| Namibia | 2013 | 567 | -0.97±1.49 | 160.9±6.3 | 171.4±7.1 | 125 | 20.1 |
| Nepal | 2016 | 1,177 | -1.49±1.44 | 151.2±5.3 | 163.7±6.1 | 438 | 36.1 |
| Sierra Leone | 2013 | 2,547 | -1.28±2.16 | 157.7±6.3 | 166.9±6.4 | 952 | 37.3 |
| Sao Tome and Principe | 2008 | 754 | -1.11±1.79 | 159.1±7.1 | 170.2±7.1 | 219 | 30.5 |
| Swaziland | 2006 | 519 | -1.09±1.49 | 158.9±6.0 | 170.4±6.0 | 143 | 28.2 |
| Uganda | 2016 | 2,611 | -1.13±1.48 | 159.3±6.4 | 169.6±7.0 | 709 | 26.7 |
| South Africa | 2016 | 206 | -1.02±1.43 | 157.9±6.0 | 170.1±6.8 | 48 | 23.2 |
| Zimbabwe | 2015 | 2,556 | -1.17±1.40 | 160.3±6.1 | 171.6±6.7 | 650 | 26.3 |
| Total | - | 50,372 | -1.37±1.72 | 154.3±6.8 | 165.7±7.4 | 17,432 | 34.5 |

HAZ: height-for-age z score.

The distribution of child HAZ, maternal height, and paternal height was expressed as Mean ± Standard deviation.

**Table S2**. Characteristics between the included and excluded participants by country.

| Country | Sample size of included participants | Sample size of excluded participants | Child sex (boy) | | |  | Child age | | |  | Maternal age | | |  | Paternal age | | |
| --- | --- | --- | --- | --- | --- | --- | --- | --- | --- | --- | --- | --- | --- | --- | --- | --- | --- |
|  |  |  | Included | Excluded | P |  | Included | Excluded | P |  | Included | Excluded | P |  | Included | Excluded | P |
| **Ethiopia** | 5457 | 4482 | 2788 (51.1) | 2282 (50.9) | 0.862 |  | 28.8±17.3 | 29.5±17.4 | 0.056 |  | 29.2±6.3 | 29.3±6.8 | 0.440 |  | 36.0±7.9 | 36.3±11.9 | 0.137 |
| **Ghana** | 1560 | 4035 | 827 (53.0) | 2078 (51.5) | 0.310 |  | 28.1±17.0 | 28.8±17.2 | 0.188 |  | 31.1±6.7 | 30.8±6.9 | 0.143 |  | 37.7±8.2 | 38.1±10.1 | 0.165 |
| India | 29478 | 218265 | 15289 (51.9) | 113320 (51.9) | 0.865 |  | 30.3±16.9 | 29.7±17.2 | <0.001 |  | 27.4±5.1 | 27.2±5.1 | <0.001 |  | 31.7±6.2 | 31.8±7.2 | 0.566 |
| **Liberia** | 1683 | 5375 | 860 (51.1) | 2732 (50.8) | 0.846 |  | 28.7±17.6 | 28.8±17.5 | 0.778 |  | 29.2±6.7 | 28.9±7.6 | 0.149 |  | 34.3±7.1 | 34.8±9.7 | 0.053 |
| **Lesotho** | 396 | 2519 | 192 (48.5) | 1244 (49.4) | 0.739 |  | 28.5±16.0 | 26.9±17.3 | 0.090 |  | 28.5±6.9 | 28.1±6.7 | 0.274 |  | 34.1±9.3 | 33.3±8.3 | 0.085 |
| **Maldives** | 861 | 2174 | 445 (51.7) | 1103 (50.7) | 0.638 |  | 30.2±16.5 | 30.4±17.3 | 0.811 |  | 30.2±5.1 | 30.1±5.5 | 0.706 |  | 33.4±6.2 | 33.9±7.5 | 0.104 |
| **Namibia** | 567 | 4251 | 273 (48.1) | 2104 (49.5) | 0.547 |  | 29.5±17.3 | 28.1±17.2 | 0.067 |  | 30.3±7.0 | 29.8±7.0 | 0.112 |  | 36.1±8.5 | 36.5±9.7 | 0.347 |
| **Nepal** | 1177 | 3643 | 614 (52.2) | 1933 (53.1) | 0.593 |  | 30.1±17.2 | 29.7±17.1 | 0.570 |  | 26.8±5.9 | 26.5±5.3 | 0.102 |  | 31.0±7.3 | 30.7±6.3 | 0.170 |
| Sierra Leone | 2547 | 8071 | 1227 (48.2) | 4041 (50.1) | 0.096 |  | 28.9±16.9 | 28.2±17.2 | 0.078 |  | 29.6±6.7 | 28.7±7.3 | <0.001 |  | 37.7±8.5 | 39.8±11.3 | <0.001 |
| **Sao Tome and Principe** | 754 | 1097 | 368 (48.8) | 558 (50.9) | 0.384 |  | 28.9±16.4 | 27.9±17.4 | 0.212 |  | 29.2±7.0 | 29.1±7.2 | 0.649 |  | 34.0±7.7 | 34.5±9.2 | 0.219 |
| **Swaziland** | 519 | 2018 | 262 (50.5) | 1016 (50.3) | 0.956 |  | 28.7±16.8 | 28.2±17.3 | 0.555 |  | 28.8±6.4 | 28.3±7.0 | 0.139 |  | 35.0±7.1 | 35.2±9.9 | 0.665 |
| Uganda | 2611 | 12022 | 1284 (49.2) | 6054 (50.4) | 0.274 |  | 28.6±17.1 | 29.4±17.3 | 0.033 |  | 28.8±6.6 | 28.4±6.8 | 0.009 |  | 34.3±8.0 | 35.0±9.3 | <0.001 |
| South Africa | 206 | 3183 | 122 (59.2) | 1615 (50.7) | 0.018 |  | 32.7±17.3 | 29.7±17.3 | 0.016 |  | 31.5±6.3 | 28.7±6.7 | <0.001 |  | 37.9±8.2 | 36.5±8.2 | 0.023 |
| **Zimbabwe** | 2556 | 3201 | 1259 (49.3) | 1568 (49.0) | 0.838 |  | 29.4±17.3 | 29.5±17.2 | 0.817 |  | 28.9±6.3 | 28.8±6.7 | 0.249 |  | 34.8±7.5 | 35.1±9.7 | 0.199 |

The proportion of boy was expressed as n (%), and the distribution of child age, maternal age, and paternal age was expressed as Mean ± Standard deviation.

The proportion of boy was compared using Chi-square test, and the means of child age, maternal age, and paternal age were compared using Student’s t test.

Country name was bolded if none of the difference of child sex, child age, maternal age, and paternal age between the included and excluded participants was statistically significant.

**Table S3. The adjusted relative risk (RR) with 95% confidence intervals (CIs) based on each combination of maternal and paternal height quintile.**

| Maternal height quintile | Paternal height quintile | | | | | | | | | | | | | |
| --- | --- | --- | --- | --- | --- | --- | --- | --- | --- | --- | --- | --- | --- | --- |
|  | First | |  | Second | |  | Third | |  | Fourth | |  | Fifth | |
|  | RR (95% CI) | P Value |  | RR (95% CI) | P Value |  | RR (95% CI) | P Value |  | RR (95% CI) | P Value |  | RR (95% CI) | P Value |
| First | 3.23 (2.83-3.68) | <0.001 |  | 2.72 (2.38-3.11) | <0.001 |  | 2.47 (2.14-2.85) | <0.001 |  | 2.20 (1.89-2.56) | <0.001 |  | 1.96 (1.65-2.33) | <0.001 |
| Second | 3.01 (2.62-3.47) | <0.001 |  | 2.50 (2.18-2.87) | <0.001 |  | 2.32 (2.01-2.67) | <0.001 |  | 1.99 (1.70-2.32) | <0.001 |  | 1.69 (1.44-1.99) | <0.001 |
| Third | 2.66 (2.32-3.06) | <0.001 |  | 2.29 (1.98-2.65) | <0.001 |  | 2.21 (1.90-2.55) | <0.001 |  | 1.81 (1.55-2.12) | <0.001 |  | 1.46 (1.23-1.73) | <0.001 |
| Fourth | 2.66 (2.31-3.06) | <0.001 |  | 2.10 (1.81-2.44) | <0.001 |  | 1.98 (1.71-2.29) | <0.001 |  | 1.65 (1.41-1.93) | <0.001 |  | 1.28 (1.08-1.51) | 0.005 |
| Fifth | 2.22 (1.91-2.59) | <0.001 |  | 1.84 (1.57-2.16) | <0.001 |  | 1.61 (1.37-1.88) | <0.001 |  | 1.39 (1.18-1.65) | <0.001 |  | 1.00 (Reference) |  |

Models were adjusted for maternal age, maternal highest level of education, paternal age, and paternal highest level of education; sex and age of the child, birth interval, and birth order; household wealth status, residence, country, and survey year.

**Table S4**. Adjusted β between maternal and paternal height and child height-for-age z score and relative risk (RR) between maternal and paternal height quintile and children stunting with their 95% confidence intervals (CIs) for sensitivity analysis.

| Group | Maternal | |  | Paternal | |
| --- | --- | --- | --- | --- | --- |
|  | β/RR (95% CI) | *P* Value |  | β/RR (95% CI) | *P* Value |
| **Additionally adjustment for parental smoking (n=45,722)** | |  |  |  |  |
| Height | 0.048 (0.046-0.050) | <0.001 |  | 0.021 (0.020-0.023) | <0.001 |
| Height quintile (within country) |  |  |  |  |  |
| First, short | 1.90 (1.78-2.03) | <0.001 |  | 1.55 (1.46-1.65) | <0.001 |
| Second | 1.60 (1.49-1.71) | <0.001 |  | 1.45 (1.36-1.55) | <0.001 |
| Third | 1.48 (1.38-1.58) | <0.001 |  | 1.31 (1.22-1.40) | <0.001 |
| Fourth | 1.25 (1.16-1.34) | <0.001 |  | 1.21 (1.13-1.29) | <0.001 |
| Fifth, tall | 1.00 (Reference) |  |  | 1.00 (Reference) |  |
|  |  |  |  |  |  |
| **Additionally adjustment for parental occupation (n=48,600)** | |  |  |  |  |
| Height | 0.047 (0.045-0.049) | <0.001 |  | 0.021 (0.019-0.023) | <0.001 |
| Height quintile (within country) |  |  |  |  |  |
| First, short | 1.87 (1.76-1.99) | <0.001 |  | 1.55 (1.46-1.65) | <0.001 |
| Second | 1.57 (1.47-1.67) | <0.001 |  | 1.44 (1.35-1.53) | <0.001 |
| Third | 1.45 (1.36-1.54) | <0.001 |  | 1.30 (1.22-1.39) | <0.001 |
| Fourth | 1.24 (1.15-1.33) | <0.001 |  | 1.21 (1.13-1.29) | <0.001 |
| Fifth, tall | 1.00 (Reference) |  |  | 1.00 (Reference) |  |
|  |  |  |  |  |  |
| **Additionally adjustment for breastfeeding initiation time and duration time (n=36,414)** | | | | |  |
| Height | 0.046 (0.044-0.048) | <0.001 |  | 0.022 (0.020-0.023) | <0.001 |
| Height quintile (within country) |  |  |  |  |  |
| First, short | 1.94 (1.80-2.08) | <0.001 |  | 1.60 (1.50-1.72) | <0.001 |
| Second | 1.61 (1.50-1.74) | <0.001 |  | 1.42 (1.33-1.53) | <0.001 |
| Third | 1.53 (1.41-1.65) | <0.001 |  | 1.30 (1.21-1.40) | <0.001 |
| Fourth | 1.27 (1.17-1.38) | <0.001 |  | 1.21 (1.13-1.31) | <0.001 |
| Fifth, tall | 1.00 (Reference) |  |  | 1.00 (Reference) |  |
|  |  |  |  |  |  |
| **Additionally adjustment for child anemia (n=43,156)** | |  |  |  |  |
| Height | 0.048 (0.046-0.05) | <0.001 |  | 0.022 (0.020-0.023) | <0.001 |
| Height quintile (within country) |  |  |  |  |  |
| First, short | 1.90 (1.78-2.02) | <0.001 |  | 1.54 (1.45-1.64) | <0.001 |
| Second | 1.59 (1.49-1.70) | <0.001 |  | 1.44 (1.35-1.54) | <0.001 |
| Third | 1.47 (1.38-1.57) | <0.001 |  | 1.31 (1.23-1.40) | <0.001 |
| Fourth | 1.27 (1.18-1.36) | <0.001 |  | 1.21 (1.13-1.29) | <0.001 |
| Fifth, tall | 1.00 (Reference) |  |  | 1.00 (Reference) |  |
|  |  |  |  |  |  |
| **Additionally adjustment for child acute respiratory infection (n=50,340)** | | |  |  |  |
| Height | 0.047 (0.043-0.052) | <0.001 |  | 0.022 (0.018-0.025) | <0.001 |
| Height quintile (within country) |  |  |  |  |  |
| First, short | 1.90 (1.78-2.01) | <0.001 |  | 1.55 (1.47-1.65) | <0.001 |
| Second | 1.58 (1.48-1.68) | <0.001 |  | 1.44 (1.35-1.53) | <0.001 |
| Third | 1.46 (1.37-1.56) | <0.001 |  | 1.30 (1.22-1.39) | <0.001 |
| Fourth | 1.25 (1.17-1.34) | <0.001 |  | 1.21 (1.13-1.29) | <0.001 |
| Fifth, tall | 1.00 (Reference) |  |  | 1.00 (Reference) |  |
|  |  |  |  |  |  |
| **Additionally adjustment for child diarrhea (n=50,311)** | |  |  |  |  |
| Height | 0.047 (0.043-0.051) | <0.001 |  | 0.022 (0.018-0.025) | <0.001 |
| Height quintile (within country) |  |  |  |  |  |
| First, short | 1.90 (1.78-2.01) | <0.001 |  | 1.55 (1.46-1.65) | <0.001 |
| Second | 1.58 (1.48-1.68) | <0.001 |  | 1.44 (1.35-1.53) | <0.001 |
| Third | 1.46 (1.37-1.56) | <0.001 |  | 1.31 (1.22-1.39) | <0.001 |
| Fourth | 1.25 (1.17-1.34) | <0.001 |  | 1.21 (1.13-1.28) | <0.001 |
| Fifth, tall | 1.00 (Reference) |  |  | 1.00 (Reference) |  |
|  |  |  |  |  |  |
| **Additionally adjustment for household drinking water source (n=49,891)** | | |  |  |  |
| Height | 0.047 (0.046-0.049) | <0.001 |  | 0.022 (0.020-0.023) | <0.001 |
| Height quintile (within country) |  |  |  |  |  |
| First, short | 1.89 (1.78-2.01) | <0.001 |  | 1.55 (1.46-1.64) | <0.001 |
| Second | 1.57 (1.48-1.68) | <0.001 |  | 1.43 (1.35-1.52) | <0.001 |
| Third | 1.46 (1.37-1.56) | <0.001 |  | 1.30 (1.22-1.39) | <0.001 |
| Fourth | 1.25 (1.16-1.33) | <0.001 |  | 1.20 (1.13-1.28) | <0.001 |
| Fifth, tall | 1.00 (Reference) |  |  | 1.00 (Reference) |  |
|  |  |  |  |  |  |
| **Additionally adjustment for parental smoking, parental occupation, breastfeeding initiation time, child anemia, child acute respiratory infection, child diarrhea, household drinking water source (n=26,858)** | | | | | |
| Height | 0.047 (0.045-0.049) | <0.001 |  | 0.021 (0.019-0.023) | <0.001 |
| Height quintile (within country) |  |  |  |  |  |
| First, short | 1.91 (1.76-2.07) | <0.001 |  | 1.58 (1.46-1.71) | <0.001 |
| Second | 1.61 (1.48-1.75) | <0.001 |  | 1.44 (1.33-1.56) | <0.001 |
| Third | 1.52 (1.40-1.65) | <0.001 |  | 1.30 (1.19-1.41) | <0.001 |
| Fourth | 1.26 (1.15-1.38) | <0.001 |  | 1.23 (1.13-1.34) | <0.001 |
| Fifth, tall | 1.00 (Reference) |  |  | 1.00 (Reference) |  |
|  |  |  |  |  |  |
| **Samples from India (n=29,478)** |  |  |  |  |  |
| Height | 0.047 (0.041-0.053) | <0.001 |  | 0.016 (0.011-0.021) | <0.001 |
| Height quintile (within country) |  |  |  |  |  |
| First, short | 1.82 (1.68-1.97) | <0.001 |  | 1.52 (1.40-1.64) | <0.001 |
| Second | 1.55 (1.43-1.68) | <0.001 |  | 1.41 (1.30-1.53) | <0.001 |
| Third | 1.47 (1.35-1.59) | <0.001 |  | 1.31 (1.20-1.42) | <0.001 |
| Fourth | 1.25 (1.15-1.37) | <0.001 |  | 1.22 (1.13-1.32) | <0.001 |
| Fifth, tall | 1.00 (Reference) |  |  | 1.00 (Reference) |  |
|  |  |  |  |  |  |
| **Samples from Non-India countries (n=20,894)** | |  |  |  |  |
| Height | 0.048 (0.043-0.053) | <0.001 |  | 0.029 (0.024-0.033) | <0.001 |
| Height quintile (within country) |  |  |  |  |  |
| First, short | 2.02 (1.84-2.22) | <0.001 |  | 1.64 (1.49-1.79) | <0.001 |
| Second | 1.64 (1.48-1.81) | <0.001 |  | 1.50 (1.36-1.65) | <0.001 |
| Third | 1.46 (1.32-1.62) | <0.001 |  | 1.30 (1.18-1.44) | <0.001 |
| Fourth | 1.24 (1.12-1.38) | <0.001 |  | 1.19 (1.07-1.32) | 0.001 |
| Fifth, tall | 1.00 (Reference) |  |  | 1.00 (Reference) |  |
|  |  |  |  |  |  |
| **Only the first birth offspring included (n=14,649)** | |  |  |  |  |
| Height | 0.051 (0.043-0.059) | <0.001 |  | 0.013 (0.007-0.020) | <0.001 |
| Height quintile (within country) |  |  |  |  |  |
| First, short | 2.10 (1.86-2.36) | <0.001 |  | 1.49 (1.32-1.67) | <0.001 |
| Second | 1.72 (1.52-1.95) | <0.001 |  | 1.41 (1.25-1.59) | <0.001 |
| Third | 1.48 (1.31-1.68) | <0.001 |  | 1.30 (1.16-1.46) | <0.001 |
| Fourth | 1.34 (1.17-1.54) | <0.001 |  | 1.27 (1.12-1.43) | <0.001 |
| Fifth, tall | 1.00 (Reference) |  |  | 1.00 (Reference) |  |
|  |  |  |  |  |  |
| **Samples from countries where the characteristics between the included and excluded participants were not different (n=15,530)** | | | | |  |
| Height | 0.047 (0.041-0.053) | <0.001 |  | 0.027 (0.022-0.032) | <0.001 |
| Height quintile (within country) |  |  |  |  |  |
| First, short | 1.98 (1.06-1.77) | <0.001 |  | 1.59 (1.06-1.42) | <0.001 |
| Second | 1.60 (1.06-1.43) | <0.001 |  | 1.47 (1.06-1.31) | <0.001 |
| Third | 1.47 (1.06-1.30) | <0.001 |  | 1.28 (1.06-1.13) | <0.001 |
| Fourth | 1.17 (1.07-1.03) | 0.013 |  | 1.18 (1.07-1.04) | 0.010 |
| Fifth, tall | 1.00 (Reference) |  |  | 1.00 (Reference) |  |

Models were adjusted for maternal age, maternal highest level of education, paternal age, and paternal highest level of education; sex and age of the child, birth interval, and birth order; household wealth status, residence, country (not for the group of samples from India), and survey year.
